# Supplementary figures and images for: Hybrid dedicated and distributed coding in PMd/M1 provides separation and interaction of bilateral arm signals
Source: PLoS Comput Biol. 2021 Nov 22;17(11):e1009615. doi: 10.1371/journal.pcbi.1009615 (PMC8648118; doi:10.1371/journal.pcbi.1009615)

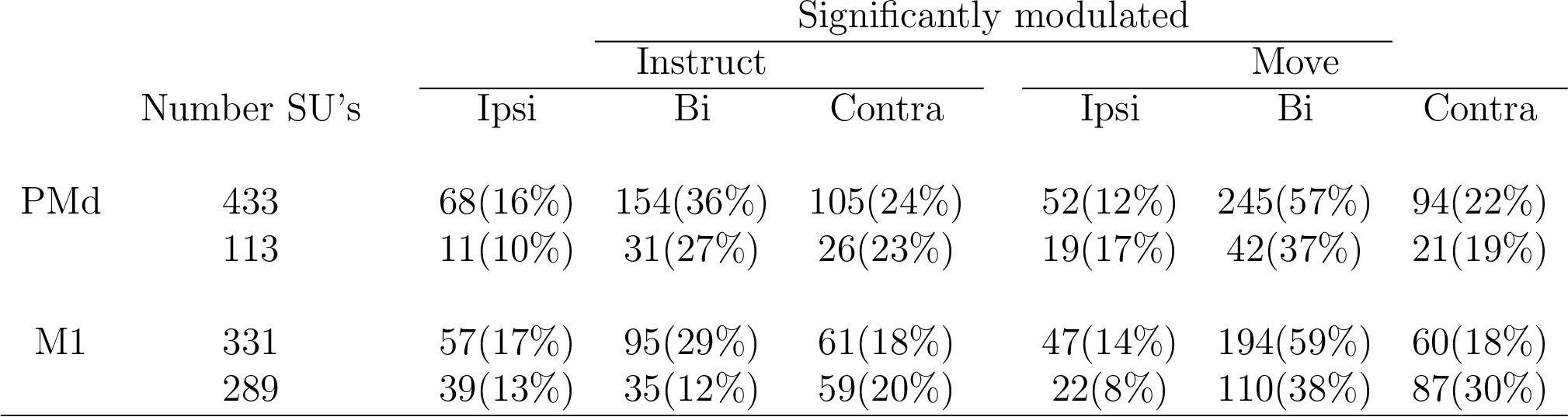

Supplement: S1 Table — For well isolated single-units in each brain area, the proportions of the total population that were significantly modulated when compared with the Rest phase (two-sample t-test, p<0.05) are displayed in each cell. For each phase, single-units were classified as uniquely ipsi, contra, or bilaterally modulated. Top row in each pair of rows represents Monkey O, bottom row Monkey W. (TIF) [file pcbi.1009615.s001.tif]

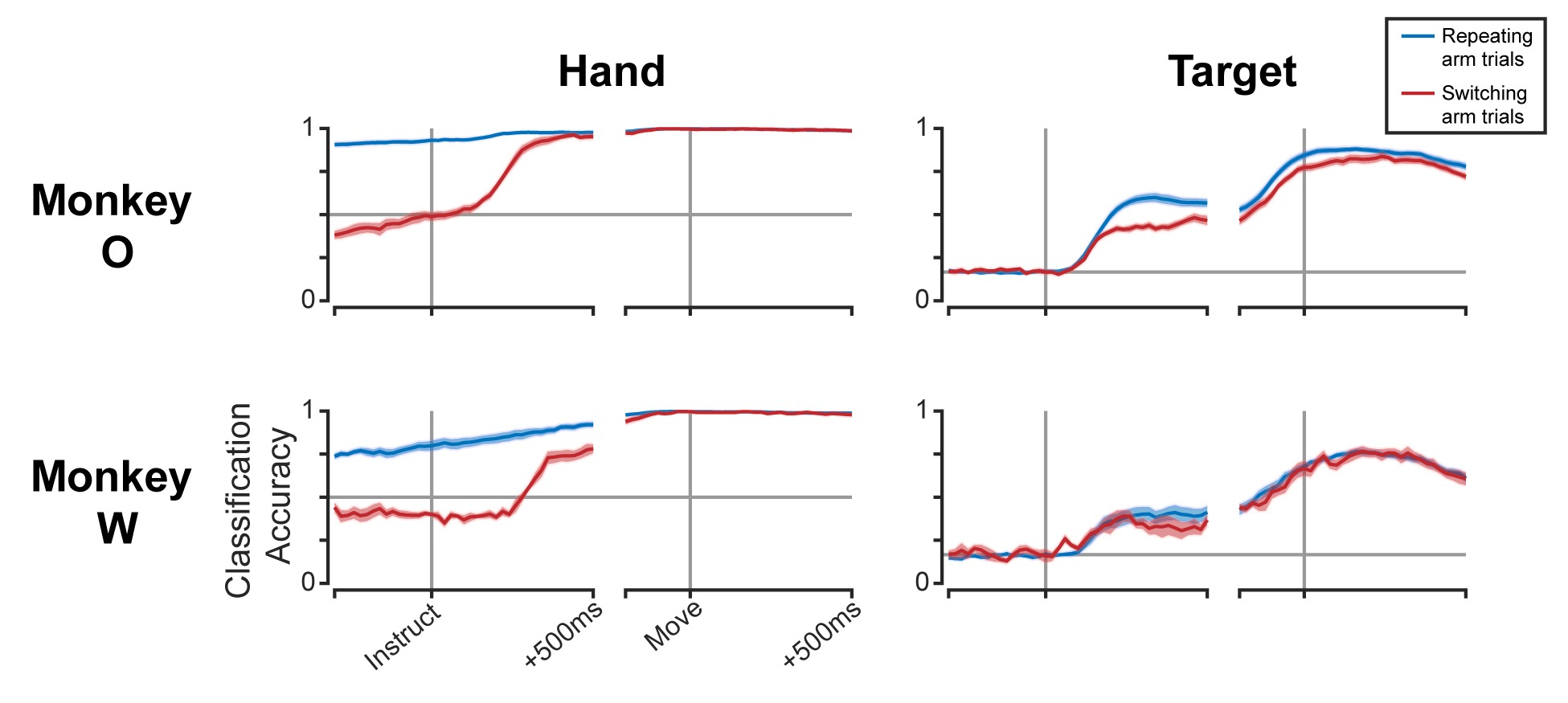

Supplement: S1 Fig — Cross-validated classification accuracy for hand (left column) and target (right column) assignments. LDA models were trained on only trials that required use of the same arm as the previous trial, then tested on either held-out repeating arm trials (blue lines) or switching arm trials (red lines). Separate models were used for each timepoint. Horizontal grey lines indicate chance level. 13 Sessions for monkey O (top row); 7 sessions for monkey W (bottom row). Mean +/- standard error across sessions. (TIF) [file pcbi.1009615.s002.tif]

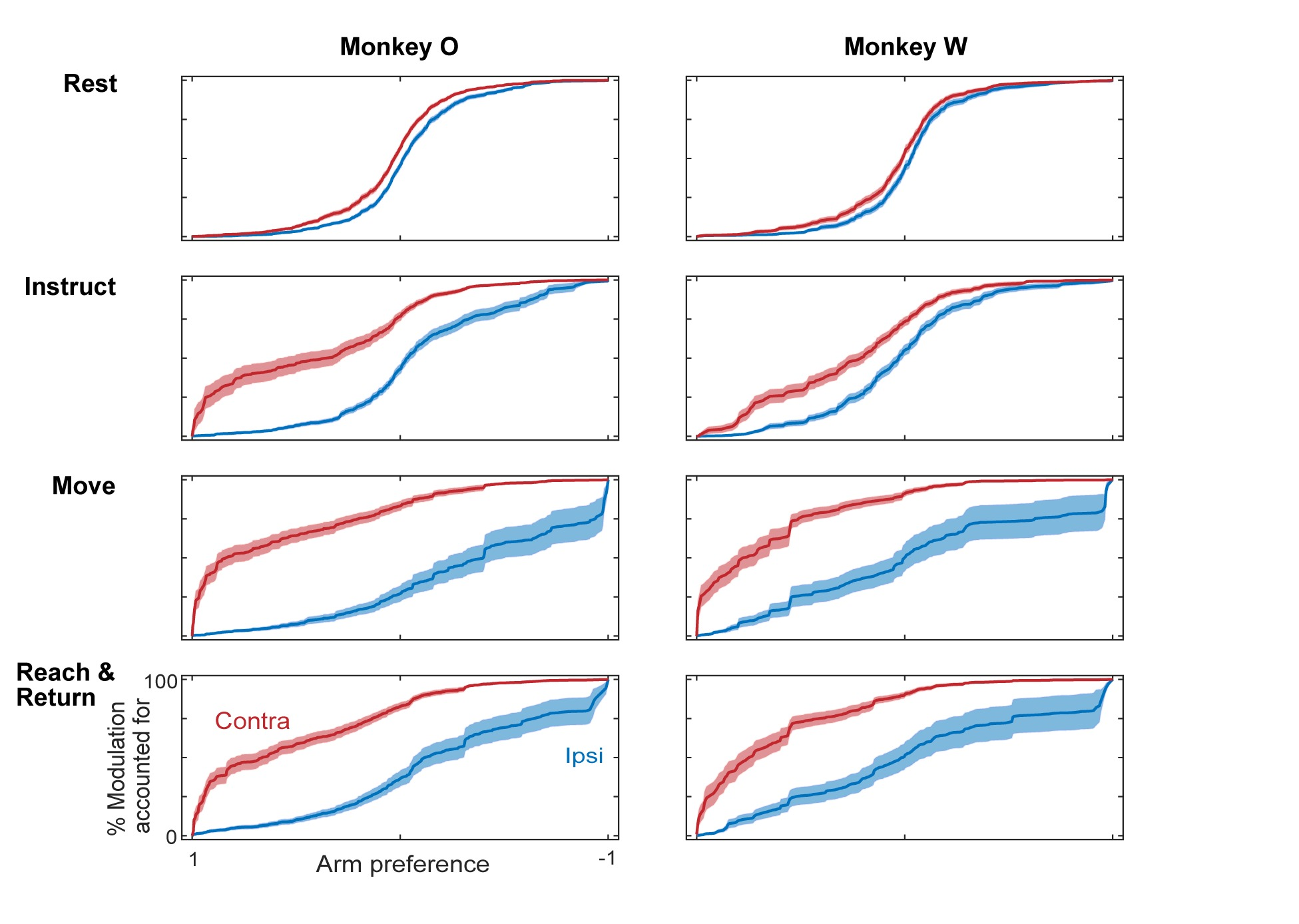

Supplement: S2 Fig — The cumulative modulation captured by units at each value of arm preference is plotted for both monkeys in each task phase. Units were first sorted according to their arm preference in an independent dataset. The cumulative modulation of ipsi- and contralateral responses was then computed at each arm preference value. Each datapoint indicates the proportion of modulation accounted for by all units with arm preference values to the left of the indexed position. Mean +/- bootstrapped standard error. (TIF) [file pcbi.1009615.s003.tif]

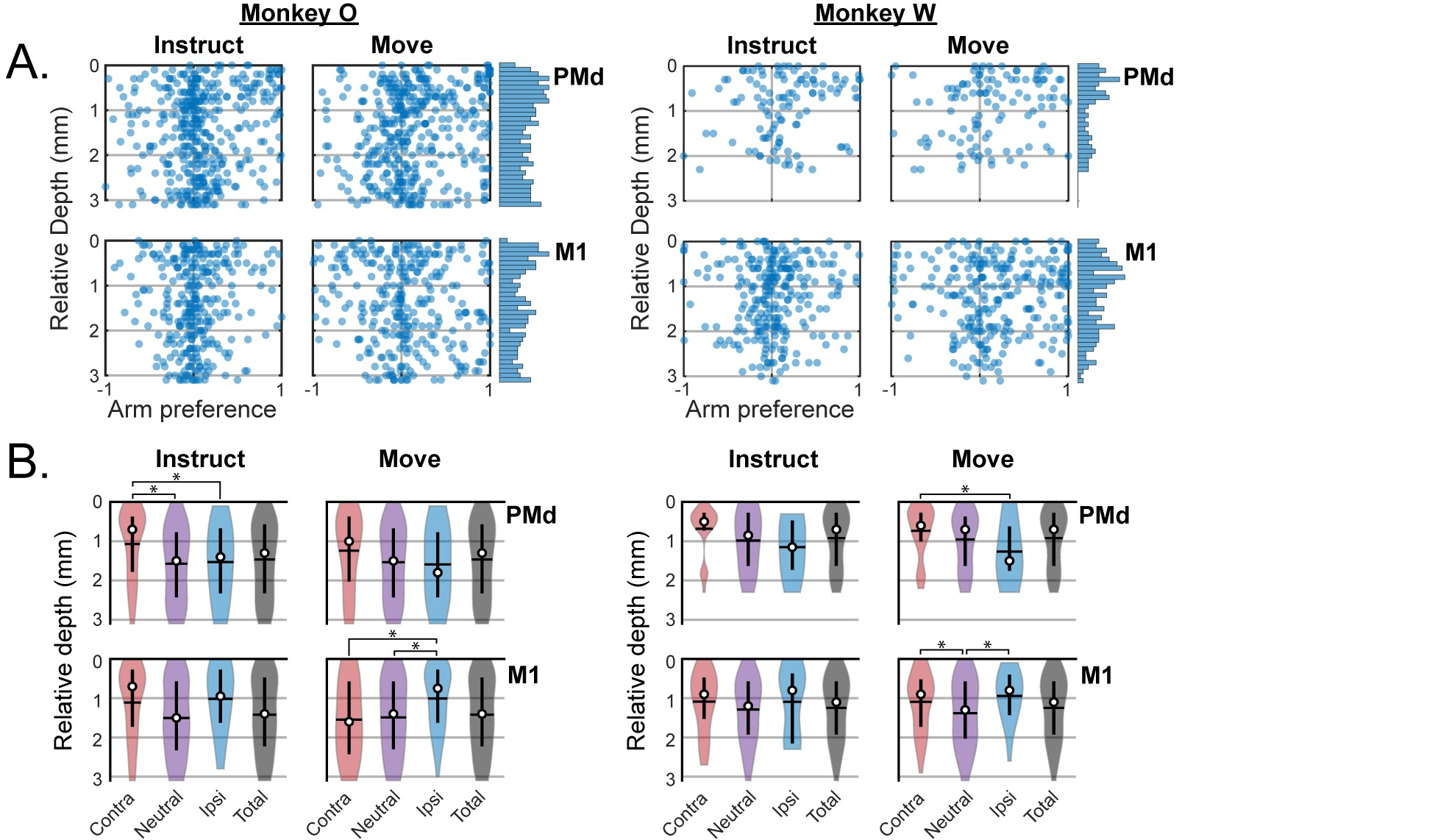

Supplement: S3 Fig — (A). The depth of the most superficial electrode was set to a value of 0, providing a reference point for the depth of all units (electrodes were spaced by 100um in a line along the length of the probe). Depth of each unit is plotted as a function of arm preference for PMd and M1 in the Instruct and Move phases. The histograms to the right display the marginal distribution of all unit depths. Recordings in Monkey W were done with 24 channel probes, except for the left M1 probe which had 32 channels. This resulted in less sampling between depths of 2.3 and 3.1mm. (B). Units were classified as Contra-dedicated, Neutral, or Ipsi-dedicated (see Fig 5C and 5F). For each category, horizontal black lines, circles, and extended vertical lines indicate the mean, median, and interquartile range. Asterisks indicate significant pairwise differences (permutation test, p<0.05) determined after an initial ANOVA for group differences. (TIF) [file pcbi.1009615.s004.tif]

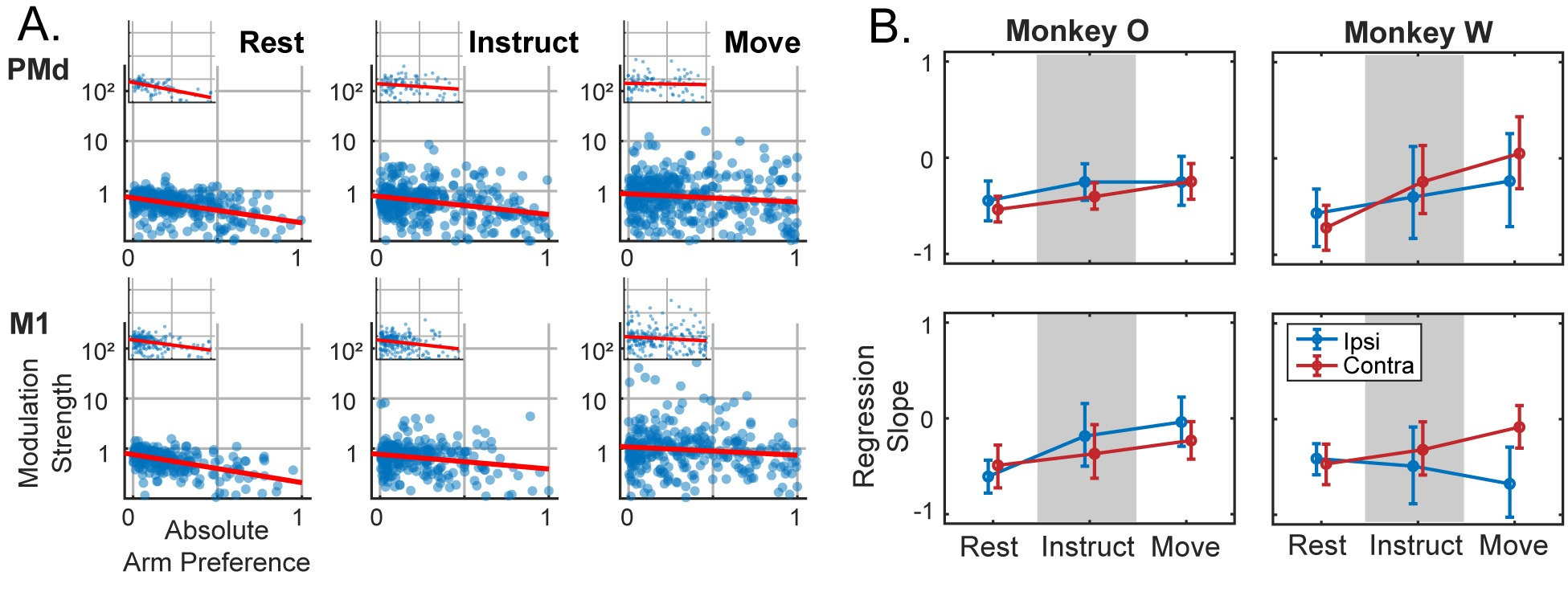

Supplement: S4 Fig — Companion figure for Fig 5A and 5B. (A) Modulation for the non-preferred arm plotted against arm preference, for all units in each brain area and task phase. Log-linear best fit lines are displayed in red. Inset figures belong to Monkey W. (B) Slopes of regression lines fit to data from (A), independently for ipsi- and contra-preferring sub-populations. Mean +/- bootstrapped 95% confidence interval. Note the different y-axis from Fig 4B. The slope was not significantly greater than 0 in any condition, meaning that increased arm preference is associated uniquely with greater modulation in the preferred arm, as opposed to an increase for both arms that is just larger for the preferred arm. (TIF) [file pcbi.1009615.s005.tif]

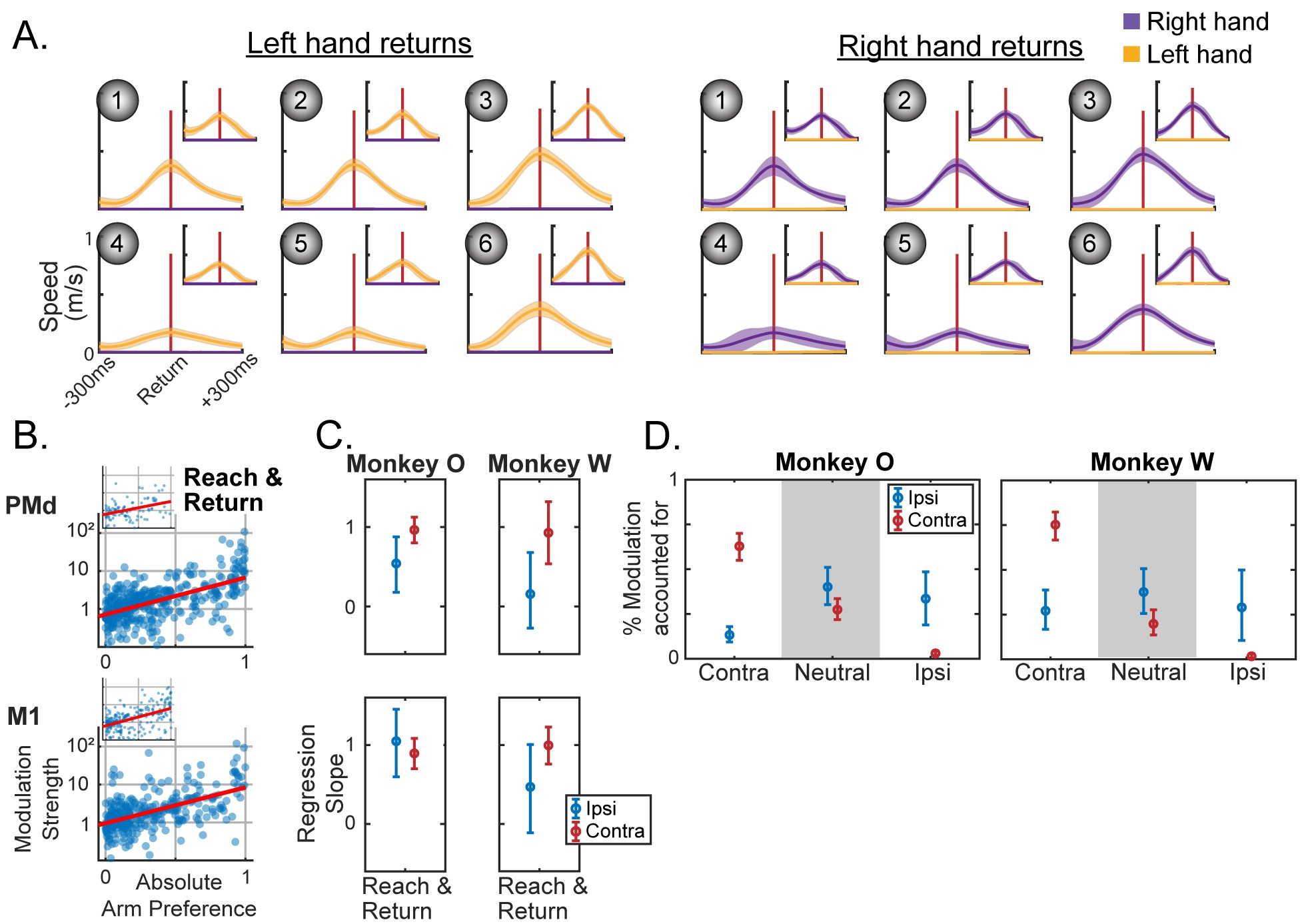

Supplement: S5 Fig — To determine whether having a limited range of reach directions was responsible for the observation of arm-dedicated signals, select analyses were performed again on data that included return movements. By including these movements, which were opposite the direction of the outward reaches used in the primary analyses, the range of sampled behavior was greatly increased. (A) Speed profiles for return movements following target acquisition during left- or right-hand trials. Individual trials were aligned to peak return speed, indicated by the vertical red line. Both reaching and stationary hands are plotted in each. Despite being unconstrained by the task, the non-selected hand remained still during the return. Monkey O main, monkey W inset. Mean +/- standard deviation. (B-D) Analyses from Fig 5 repeated using Move phase data concatenated with 300ms of data beginning 200ms before the point of peak return speed, i.e. reach and return. (B) Compare to Fig 5A. Modulation for the preferred arm plotted against arm preference, for all units in each brain area. Log-linear best fit lines are displayed in red. Inset figures belong to Monkey W. (C) Compare to Fig 5B. Slopes of regression lines fit to data from (B), independently for ipsi- and contra-preferring sub-populations. Mean +/- bootstrapped 95% confidence interval. (D) Compare to Fig 5F. The proportion of modulation within each partition from (C) during ipsi- or contralateral movements. Note that the total modulation is significantly lower for ipsilateral movements, particularly for Monkey W, and these data are only displayed as proportions. Mean +/- bootstrapped 95% confidence interval. (TIF) [file pcbi.1009615.s006.tif]

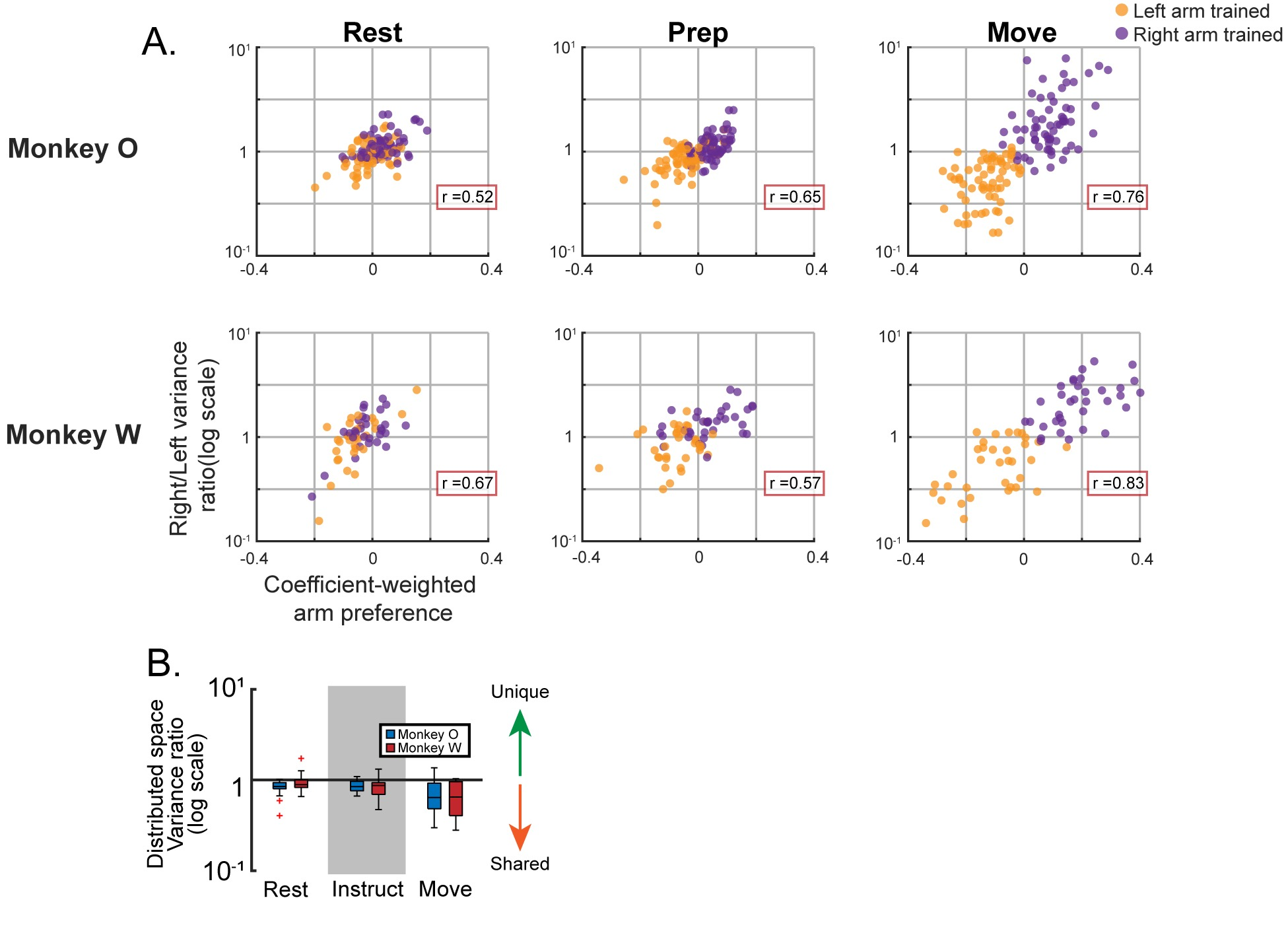

Supplement: S6 Fig — Prior to performing PCA, an alternative method of normalizing firing rates was used for these plots. Rather than dividing by the standard deviation at Rest, each unit’s firing rate trace was divided by the full firing rate range + 5Hz [10,11,22]. This will mitigate the effect of highly modulated units, which PCA will preferentially represent otherwise. (A) Repetition of Fig 7D. (B) Repetition of Fig 8F. (TIF) [file pcbi.1009615.s007.tif]

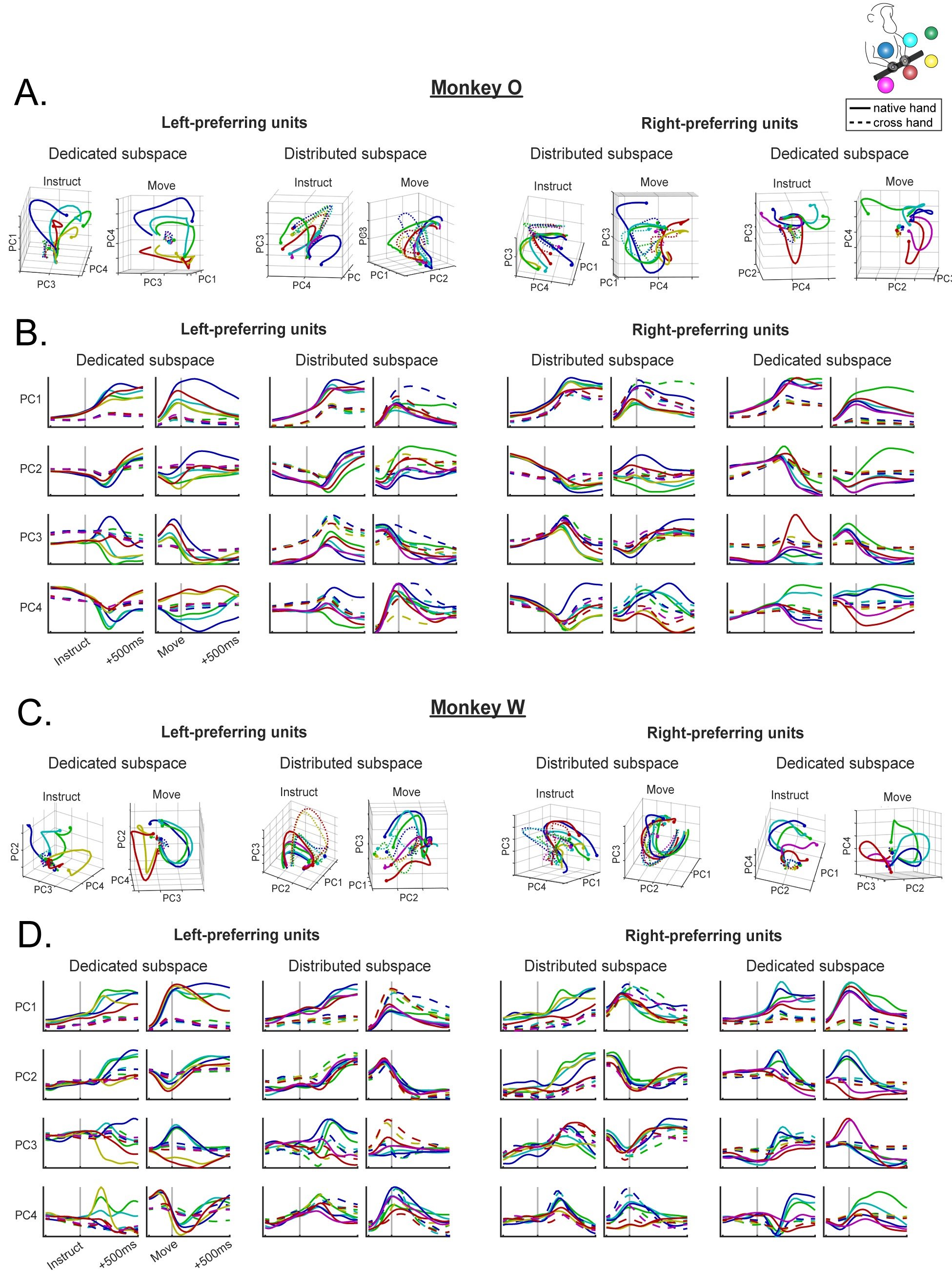

Supplement: S7 Fig — A-B. Projections for monkey O. A. 3D projections of activity from neural sub-populations partitioned based on their preferred arm (see Fig 8A). Each PCA model was trained using trials where the reaching hand was either the preferred hand (dedicated subspace) or the non-preferred hand (distributed subspace) of the sub-population. Separate models were trained during the Instruct phase (300ms before to 500ms after instruction onset) and the Move phase (200ms before to 500ms after instruction onset). The models were trained on trial-averaged data for each target to provide a single visualization for each group. Each target trace is color coded according to the cartoon in the upper right. The projected data is from an independent validation set that included only 5 of the 6 targets. Solid lines indicate data using the same hand as the training set (native hand), and dashed lines indicate the opposite hand projections (cross hand). B. The data from A plotted against time for the top 4 PC’s. C-D. Projections for monkey W. (TIF) [file pcbi.1009615.s008.tif]

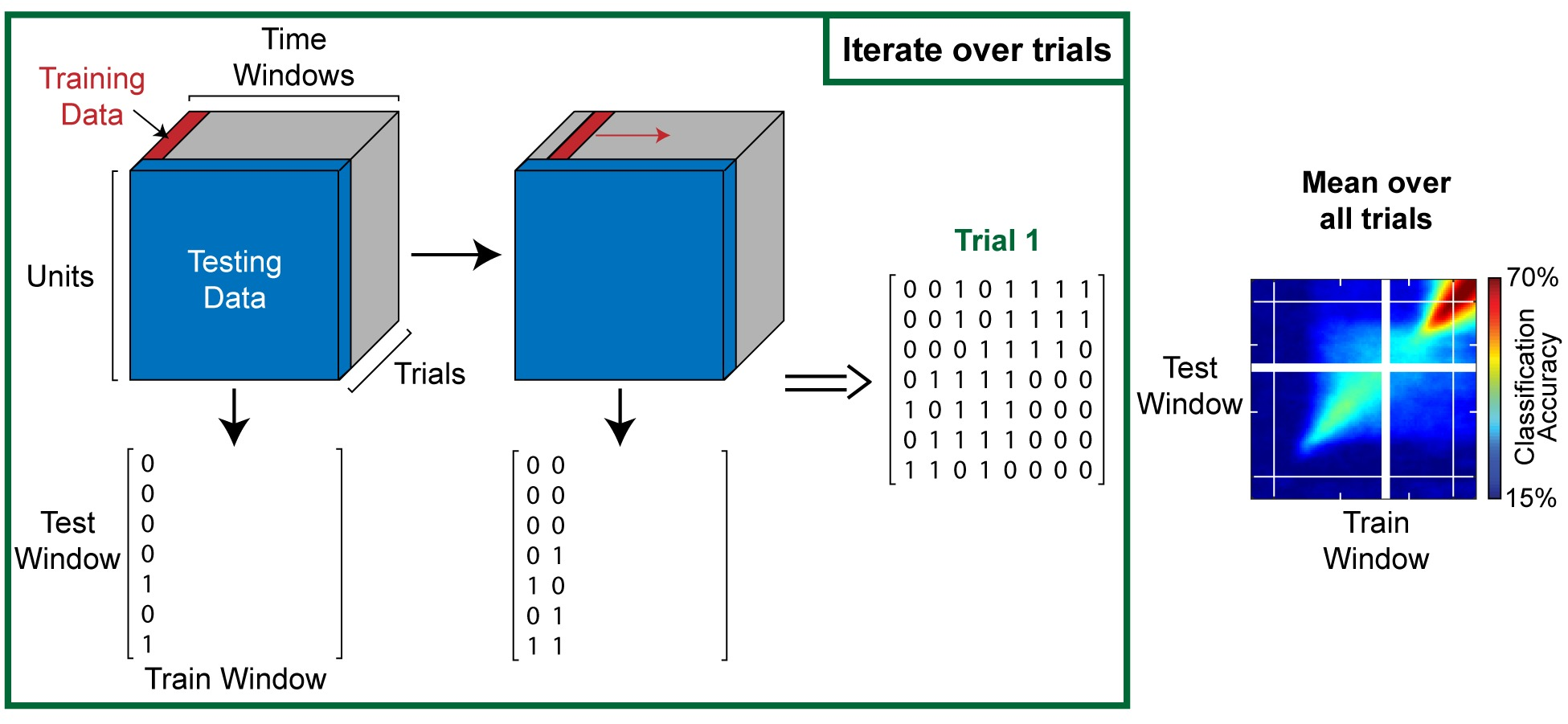

Supplement: S8 Fig — This schematic outlines the process for fine timescale analysis of population coding using LDA and leave-one-out cross-validation. Neural data were organized as 3D tensors (units, time windows, trials). Models were trained to predict targets using a single time window and all but one trial. Those models were then used to predict the target on the held-out trial, making separate predictions based on neural data from each time window. The process was then repeated using the next time window as training data until all possible pairs of time windows had been used as training and testing data. This constituted a 2D matrix of “hit” booleans (number time windows x number time windows) for the predictions of a single trial. After iterating over all trials to be used as held-out test data, the mean was taken across trials to construct a single 2D matrix of classification accuracy. The same basic process was used for visualizing the development of subspace separation, but instead of leave-one-out cross-validation trial sets were repeatedly divided into two random halves of equal size. Covariance alignment was then computed between all possible pairs of timepoints for the two disjoint trial sets. (TIF) [file pcbi.1009615.s009.tif]
