# Supplementary material for: Hybrid dedicated and distributed coding in PMd/M1 provides separation and interaction of bilateral arm signals
Source: PLoS Comput Biol. 2021 Nov 22;17(11):e1009615. doi: 10.1371/journal.pcbi.1009615 (PMC8648118; doi:10.1371/journal.pcbi.1009615)
Supplement: Attachment — Submitted filename: ploscb_response_letter.docx [file pcbi.1009615.s010.docx]

Dear Drs. Faisal and Marinazzo,

We thank you and the reviewers for the very insightful comments, which we believe have helped to greatly improve the manuscript. We provide itemized responses to each comment below, along with the reviewers’ comments. The most significant changes are a new section in the Discussion that considers our results in the context of postural control, and the addition of Figure S3 describing the relationship between depth and arm preference of recorded neurons.

We look forward to your evaluation.

Sincerely,
Tanner Dixon

***********************

**The original reviewer comments are listed below with our responses and revisions described in the red text. Referenced line numbers refer to the “clean” copy of the manuscript submission.**

# **Reviewer #1: Summary:**

Monkeys made unimanual movements of the contralateral or ipsilateral arm to 6 targets (two horizontal rows of targets at the Left, Midline, and Right, one above the other), in a pseudo-randomized-block sequence in an instructed-delay task paradigm. Multiple sets of simultaneous neural recordings were made in M1 and PMd using multi-port harpoon electrodes over several daily recording sessions. Neural data were subjected to quantitative analyses including PCA-based dimensionality reduction, covariance alignment and linear discriminant analysis. The authors identified two main discharge components that they called “dedicated” and “distributed”. The “dedicated” component accounted for most of the variance, segregated activity into neurons that were mainly contralateral- or ipsilateral-arm preferring, emerged first in PMd during the Instruction period, increased further during movement, and was stronger in the contralateral hemisphere. In contrast, the “distributed” component was much weaker, did not discriminate the laterality of single-neuron activity at any phase in the trial, and was distributed across both hemispheres. The results suggest two independent functional layers or modes of operation of the motor cortical neural network. The authors proposed that the “distributed” component might be more implicated in bilateral preparatory computations and in ongoing internal processing within the network that performs certain computations that are not directly related to output control signals, while the “dedicated” component might be more implicated in generating unilateral output signals in the hemisphere contralateral to the moving arm.

## **General comments**:

This study looked for population-level neural correlates of the control of contralateral versus ipsilateral arm movements in an analytic space of distributed patterns of neural discharge variance and covariance, and in extracted orthogonal dimensions of variances in a PC space. The neural data and analyses are a novel and valuable addition to the literature on the control of arm movements. However, **where the manuscript falls short in my opinion is when it interprets the findings in terms of a control problem in a nearly equally abstract and frankly simplistic “engineering” space of ipsilateral versus contralateral arms and target locations/trajectories**. It fails to take into account that those neural circuits are trying to implement those actions by **controlling a complicated non-linear skeletomotor plant**. The authors need to think through the control problem in more biologically- and anatomically-correct terms. The following is a disorganized set of comments about this very interesting study.

## Specific comments:

**Reviewer #1, comments 1-3 are collectively responded to below comment 3.**

1. Somatotopy still matters: The neural data were collected in nominally “arm-related” regions of M1 and PMd (Figure 2), but it is entirely likely that the neurons recorded in any given day and across days were a diverse group bearing different degrees of coupling to motor outputs of different parts of the arm and body, ranging from the wrist and hand, to the shoulder, shoulder girdle and axial structures. The Willet et al. (2020) study brings this issue into clear focus. The authors did not attempt to characterize the output relationships of each recorded neuron. I’m not criticizing them for that, because I can imagine how challenging that would be. However, this issue is not raised at all in the interpretation of their findings. If a given neuron shows a clear preference to contralateral over ipsilateral arm movements or vice versa, **the functional significance of that response property will surely be different if it turns out that it is primarily coupled to wrist movements in one arm that are unlikely to be evoked during movements of the other arm, or to shoulder or axial structures that are much more likely to be activated during movements of both arms** (see next comment). Likewise, if their contra-preferring versus ipsi-preferring neurons were non-uniformly biased to more distal versus more axial body structures, respectively, that would have a significant impact on the functional implications of the response differences that they documented. The manuscript looks for patterns in an abstract space of neural discharge variances without taking into account that different neural sub-populations are very likely primarily controlling the actions of different parts of a complex skeletomotor plant, beyond the dichotomy between contralateral and ipsilateral arms. The authors need to address the limitations of interpretation resulting from this uncertainty in the nature of their neural data sets.
2. The (ground) truth is out there: It resides in biomechanics and muscle activity. This follows up on the previous comment. The authors are confronted with the challenge of interpreting the functional meaning of neural activity evoked while the monkeys performed movements using the arm contra- or ipsilateral to the recording site. They conclude that their results “suggest that ipsilateral signals are involved in some form of bilateral control” (lines 632-633) and speculate about how this might contribute to the control of the ipsilateral arm by “mirror symmetric activation” (line 651) and/or in bimanual coordination (lines 636-677). However, **the statement in lines 632-633 may only be correct at the phenomenological or descriptive level but not at the mechanistic or anatomical level**. The reason is that those speculations seem to fail to take into account what else the monkeys are doing while making the unilateral arm movements. Unless the monkeys were encased in perfectly form-fitted body casts (which might still not eliminate this problem), all of the movements of each arm will be accompanied by **biomechanically-linked bilateral adjustments of the shoulder girdle and axial structures**, and even in the ipsilateral arm. These changes would be caused by widely distributed bilateral changes in muscle activity, along with muscle activity that stabilizes different parts of the body against the perturbing effect of the arm movements but does not result in measureable movements. **This is inevitable, and would probably be most pronounced in reaching movements of one arm to targets on the other side of the body mid-line**. As a result, an undetermined amount of the neural activity recorded during ipsilateral arm movements could result from those biomechanically-linked changes in the contralateral body and arm, rather than being a signal used to control the moving ipsilateral arm itself or some unspecified form of bilateral control. This could also explain an undetermined amount of the deviation of single-neuron arm preference measurements from the theoretically “ideal” extremes of exclusive coupling to only the contra- or ipsilateral arm. **Measuring how much the hand of the non-moving arm does or does not move is a completely inadequate control for this** (lines 135-136; 159-162). **I did not see anything in the manuscript that addressed “any reasonable impacts these small movements have in our neural analyses”** (lines 164-165) to my satisfaction. I also don’t find very convincing the argument that arm-specific segregation peaked during the movement phase reduces “any concern that small movements of the non-selected arm had an impact on our results or conclusions” (lines 611-613). I don’t see how their analytic methods can differentiate activity recorded during ipsilateral arm movements that is specifically directed at ipsilateral body targets or alternatively contributes to all the biomechanically-linked adjustments and compensations of the contralateral body during ipsilateral arm movements. If I’m wrong, it would be important to prove that I’m wrong in the manuscript.
3. Perhaps the only experimental approach currently available to deal with this major confound directly while recording neural activity are the heroic techniques that Paul Cheney, Eb Fetz, Roger Lemon and others have used to demonstrate the direct causal effects of spiking activity on peripheral musculature during various tasks. Nevertheless, the authors could do something extremely valuable to start to get a handle on this issue. They could do a thorough study of EMG activity in a number of distal and proximal arm muscles as well as muscles acting on the shoulder girdle, scapula and para-axial structures, while the monkeys made contra- and ipsi-lateral arm movements. It is actually not that difficult to do with percutaneously implanted electrodes or patch electrodes applied to the skin. Paul Cheney’s work gives clear guidance on how to go about it. Ideally, this should be done on the monkeys that participated in this study, if they are still available. They could then process the EMG data in the same manner as the neural data, and look for response patterns in the n-dimensional EMG activity space that are shared with the neurons. Even more informative would be response patterns that are expressed in the neural populations that cannot be extracted from the muscle activity. That would provide a powerful clue into the neural activity that is implicated in arm movement control but cannot be accounted for by biomechanics and muscle activity. This would provide very important experimental data to assess and ultimately justify the validity of such speculations as the statements that “bilaterally distributed networks contribute to computations that do not directly represent the output” (lines 683-684) and “distributed signals could serve to enforce internal dynamics of the overall population” (lines 692-693). **There are no solid grounds for those speculations until they can demonstrate that some components of the neural variance patterns are not also seen in the muscle activity at the periphery**. If they want to make such speculations, they also need to **acknowledge this limitation of interpretation of their neural results**. I am not arguing here that the neural data in this manuscript are unpublishable without the EMG data or even that they cannot offer those interpretations until these EMG studies are done. However, I am saying that this issue must be acknowledged and addressed as a factor that limits the interpretation of the present findings. I would also strongly encourage the authors to take on this EMG study, and present a follow-up report of the implications of any similarities and differences in the neural and muscle data. That would be a positively brilliant addition to the field.

**Response to comments 1-3:**

Thank you for raising this issue – the discussion of biomechanical linkages between the arm and torso have largely been ignored in the reaching literature, but we agree that consideration of the arm as one part of a unified skeletomotor plant is important for interpreting our results. We have added a section to our Discussion (lines 647-696), asking how the activity we observe might be related to postural factors.

The statements in the original manuscript submission about small movements in the non-performing arm not impacting our results (original lines 611-613) were meant to be directed at the “dedicated” component, which was exclusively coupled to a single arm. We recognize that this was poorly communicated and have revised that statement (lines 711-713).

As noted by the reviewer, measuring kinematics of the non-performing hand is not sufficient to dismiss the possibility that biomechanically linked adjustments in the opposite hemibody could contribute to ipsilateral cortical activity and the signal component we refer to as “distributed”. While recording EMG in the non-performing arm would provide useful information, a comprehensive analysis would require EMG from many other muscles.

Because of the unrestrained behavioral setup that we used, it was difficult, if not impossible, to safely record EMG signals from the arms and axial musculature. We are also not able to record additional data in these study animals. However, we have examined the results of other studies that have recorded EMG in more constrained 2D reaching paradigms. Most of these studies have only recorded from the muscles of the arm and shoulder girdle. From our review of the literature, Cisek et al., 2003 performed the most comprehensive recordings of EMG during movements of either arm. We now discuss our results under the assumption that similar activity patterns are also present in our behavioral dataset.

We provide an explicit statement about the absence of EMG in our dataset and how that limits our interpretation (lines 667-668).

We note here that we are in the process of preparing another manuscript that addresses the effect of postural changes in the stationary arm on ipsi- and contralateral signals. The posture of the stationary arm introduces changes to the whole-body skeletomotor plant without directly changing what the reaching arm is doing. Some of these topics will therefore be more thoroughly addressed in that work.

**Reviewer #1, comments 4-5 are collectively responded to below comment 5.**

4. Bilateral signals (lines 635-654): “…correlations between the tuning for ipsilateral and contralateral arm movements in M1 units tend to be weak or absent….Cisek et al., 2003….Our results are therefore consistent with the functional hypotheses of ipsilateral cortex involving mirror symmetric activation.” I believe that your citation of Cisek et al 2003 is correct for their M1 data. However, if I’m not mistaken, one of their main findings was not just that many more PMd neurons than M1 neurons discharged during movements of both arms, but more specifically that many PMd neurons had similar directional tuning during the instructed delay period of their task independent of the arm used, suggesting an effector-independent representation of the intended motor act in a fixed external spatial reference frame. That would contradict the conclusion in the second quoted sentence about mirror symmetric activity. Of course, Cisek’s findings apply only to their delay period activity in PMd. It is entirely possible that when pooled over the entire population in both PMd and M1, the data extraction techniques used in the present study do reveal a neural response pattern that expresses properties of ipsilateral arm movements that are mirror-symmetric to the predominant contralateral movements. That would be a very cool finding. It’s a pity that the way that they present their data does not allow us to visualize their findings in a way that we can more easily relate to the motor outputs of the tasks, namely the reach movements of each arm to the 6 spatial targets.
5. With that last comment in mind, it would be extremely interesting and useful if the authors could generate “neural space trajectories” of the contralateral-arm and ipsilateral-arm neural data collected during the 6 reaching movements from the dedicated and distributed response components using the 3 most “informative” of their top 4 PCs, similar to what Ames and Churchland (2019) did for their bilateral arm data, and what Shenoy, Churchland, Yu and others have used in a number of previous studies. I know that these displays cannot replace the rigorous quantitative tests that the authors used in this manuscript to establish the statistical validity of their findings. Nevertheless, they complement those analyses by presenting the data in a way that is more graphically intuitive. A pretty picture really is often worth more a thousand words, especially if it is easier to grasp intuitively than a thousand words of descriptions of analyses and statistical test results.

**Response to comments 4-5:**

A major finding of Cisek et al., 2003 was that PMd neurons tended to have similar directional tuning for both arms during the instructed delay period of their task. We now cite this finding directly in lines 742-743. Our intent for this section was to survey correlation patterns reported in previous studies, both in extrinsic and intrinsic reference frames. Our study describes a subspace in which activity for both arms resides, a subspace that could result from any form of correlated activity for the two arms (lines 752-754).

In response to comment 5, we now include “neural space trajectories” of the data projected onto the dedicated and distributed subspaces (Figure S7). To minimize the number of plots, these are performed on trial-averaged data that is pooled across recording sessions. This is the only location in the manuscript where trial-averaging was done prior to performing PCA.

As with our response above to comments 1-3, the issue of reference frames will be addressed in our second manuscript, where we manipulate the starting locations of the two arms to more directly assess tuning properties for the two limbs.

6. Figure 4: This figure provides the summary stats of the neural responses in the task. However, they are based on units of variance, so it is not easy to grasp the meaning of these data distributions in terms of actual neural activity. It is essential to provide concrete examples so that those readers who are still interested in such things, like me, will be able to make that transformation. It would help if the authors would do the following. In Figure 3, add the activity from the Rest phase to the single-neuron plots, and display the time windows for data analysis in each sub-plot. Provide the contra and ipsi modulation strength values for the data in each window for each neuron, and its signed arm preference score for each phase. Then add to Figure 3 the mean M1 and PMd population response histograms for all 3 trial phases for each arm (similar to what they did in Figure 8B), and the resultant population modulation strengths and arm preference scores. Do that separately for the PMd and M1 populations in each monkey. Those additions would be very helpful to understand what the curves in Figure 4 mean, as well as the log scale of Figure 4A. For instance, the Rest phase plot might seem pointless because it will likely be nearly at zero, but will still be useful because the curves in the figure make it look almost the same as the Instruction and Move activity, which is almost certainly misleading. Of course, if you think that the brain is just a gigantic n-dimensional matrix of discharge variances and covariances or orthogonal dimensions in neural activity space, I guess it really doesn’t matter, right? ϑ

**Response to comment 6:**

All of the changes in this comment have been made to Figure 3. Each phase window is labeled with horizontal bars above the plots. Modulation values in each phase are added as annotations on the single-neuron plots. Signed arm preference values for Instruct and Move are included in the figure caption. The modulation strength and arm preference values for the population plots are omitted since the entire distributions are plotted in the following Figure 4. We believe these changes have made the modulation and arm preference metrics more interpretable.

**Reviewer #1, comments 7-8 are collectively responded to below comment 8.**

7. Figure 4: Wouldn’t the data in Figure 4B be easier to understand if they were plotted as simple frequency histograms rather than cumulative frequency histograms? Does Figure 4C add anything that is significantly different from Figure 4B? I’m not convinced that it does.
8. Lines 213-231 and Figure 4: The main take-home message of this text and the figure is that there is a shift to increased contralateral response correlations from Rest to Instruction and from Instruction to Move. This might be illustrated much more effectively if they plotted the frequency distributions of single-neuron CHANGES in arm preference scores between the phases, rather than the cumulative frequency scores in each phase. They could plot those distributions instead of the plots in Figure 4C, which I do not find particularly useful.

**Response to comments 7-8:**

It is difficult to determine whether histograms or cumulative distribution functions are more informative for these data since the cumulative distributions make the quantiles readily available to the reader. Plotting multiple datasets on the same axes and making quantitative visual comparisons between them is also easier with cumulative distribution plots. However, in the case of arm preference data, the histograms provide a better visualization. As a solution, we have replaced the cumulative distribution functions in Figure 4B with a collection of histograms displaying the same data.

We have also removed Figure 4C. We did plot the distribution of arm preference changes as a potential replacement, but ultimately decided to exclude these graphs because they did not seem to add much value on top of the standard histograms.

9. Lines 195-196: “….both monkeys entered arm-specific population states during the Rest phase”. Since the activity during the Rest phase is often very low and is quantified in variance units, a change from a mean of 1sp/s to 2sp/s might be highly significant and yield a high arm preference score. However, is that anywhere near as meaningful as a difference between 20sp/s and 40sp/s for ipsilateral and contralateral arm movements respectively during the Instruction or Move phases, even though it might yield a similar arm preference score? Mathematically, the statement is correct, but is that finding functionally important? Provide more justification.

**Response to comment 9:**

We do believe that the differences observed during the Rest phase are relatively minor, as compared to the differences observed during the Instruct and Move phases. However, the differences in single unit firing rates during Rest are useful for understanding why there is already some subspace separation before instructional cues were given (Figure 6). We have clarified this in lines 245-247.

10. Lines 115-117: “…we did observe target-specific information….the subspace containing this information did not segregate signals for the two arms at any stage”. The implications of those statements are unclear. Does this mean that neural activity that expresses differential signals for the 6 targets is confined only to the smaller “distributed” component (e.g., lines 488-493, 560), but is not expressed in the much larger “dedicated” component? The only mentions of target-specific signals that I could find in the manuscript relate to the “distributed” component. Does that mean that the “dedicated” component that accounts for the largest amount of variance discriminates which arm to use, but not what to do with it? If that conclusion is true, that is really surprising and counterintuitive given that at least 1000 studies starting with Evarts has shown the powerful effect of the direction of motor output on M1 and PMd activity. All of those studies would predict that a massive amount of variance should be accounted for by direction and should be coupled to the moving arm. If my conclusion isn’t true, then there is something that I don’t understand about those statements. Which is it? If the former is true, the authors will have to discuss the implications of that surprising finding in depth. If it is not true, then the authors have got to reword those sentences to avoid the confusion.

**Response to comment 10:**

We did not mean to imply that target-specific information is only in the Distributed component. We have made this more clear in lines 115-117 and elsewhere.

11. PCA analysis: The entire neural data set comprised sub-sets of simultaneously-recorded neurons with each sub-set recorded in a different daily recording session. It is not clear if they did all their PCA analyses separately only using the neural sub-sets collected simultaneously in each daily recording session, and then somehow pooled across daily sessions. Alternatively, did they do their PCAs by post-hoc pooling of all the neural data collected in different recording sessions into one large data matrix?

**Response to comment 11:**

The data were not pooled across sessions with one exception, the new supplementary Figure S7. We now make an explicit statement of this in the Results (lines 408-409).

12. Equations: Please number all equations in the manuscript sequentially.

**Response to comment 12:**

This has now been corrected.

13. Expectation: What is the Expectation term “E” in the equation in line 819? The explanation in line 820 does not help. Why change the equation from time “t” in line 801 to “phase” in line 819?

**Response to comment 13:**

We now define this term in lines 933-934 – it is the mean. We write it this way to make it recognizable as variance (expectation of squared deviation) with respect to the Rest phase mean.

Eq. 1 shows the metric as computed for a single timepoint, whereas Eq. 2 shows the same metric computed for a collection of timepoints (i.e., a phase), rewritten in a form that makes the relationship with variance more explicit.

14. 16 data samples (line 815 and elsewhere): If the phase window for data analysis is 300ms and is divided into successive non-overlapping 20ms time bins (lines 796-797), how does that yield 16 data samples per analysis period? My old but still trusty calculator tells me that 300/20=15. Are they actually calculating the instantaneous discharge rates at each 20ms window edge, starting at 0ms, rather than calculating the mean activity within each 20ms window?

**Response to comment 14:**

Correct, this is what was done. This has been clarified in the Methods (lines 910-911 and elsewhere).

15. Figure 4, 5A, 6, 8C,D, S2A: Why did they decide to de-emphasize the data from monkey W by making all of its plots little inserts in the corner of the plots for monkey O? Why not keep the plots the same size for both monkeys? They don’t even plot the corresponding data for monkey W in Figure 5C-E. What do the authors have against poor monkey W? ϑ

**Response to comment 15:**

Key summary plots are presented in the same size for both monkeys; in a few places, we opted to display plots for monkey W as insets to save space. Figure 5C-E was included to provide a visualization of the entire distribution for monkey O’s Move phase data. We have now included the equivalent plots for both monkeys and all 3 phases in Figure S2.

16. Dimensionality estimation using cross-validated data reconstruction methods (lines 371-374, and 859-890): Are the authors suggesting by such statements as “this is an essential step to avoid drawing conclusions on noise-dominated dimensions” and “they are prone to over-fitting, which include meaningless components” that the findings of the many PCA-based dimension-reduction studies that did not use those rigorous data processing steps are suspect, if not incorrect?

**Response to comment 16:**

The appropriateness of dimensionality estimation is dependent on the follow-up analyses and planned hypothesis testing. Certain analyses, like ours, require a rigorous dimensionality estimation technique. Analyses will differ in terms of their sensitivity to the number of dimensions used so the proper determination of this model hyperparameter will depend on what you are going to use the model for. We have included some discussion on this in the Methods (Dimensionality estimation section, lines 1004-1007).

17. Lines 252-253; 424-428: Explain in simple layman’s terms how neurons with similar discharge rates for movements with both arms can still make different contributions to population-level representations and thus contribute to subspace separation.

**Response to comment 17:**

The revision now includes a two-neuron example to provide an intuitive sense of how neurons with similar discharge rates for movements of either arm can still make different contributions to the population-level representations and thus contribute to subspace separation (lines 271-275).

18. Figure 7D: the label “Prep” refers to the Instruction phase? If yes, why change the label in that figure?

**Response to comment 18:**

This has now been corrected.

# Reviewer #2: Summary:

This paper, by Dixon and colleagues titled “Hybrid dedicated and distributed coding in PMD/M1 provides separation and interaction of bilateral arm signals” seeks to understand the nature of bi-hemispheric signals present in PMd and M1. The authors found one each of dedicated and distributed components. They show that the former is primarily responsible for the traditional contralateral arm control at the single unit level, whereas the latter does not discriminate between arms.

I think the paper is well-written, and the analyses are well-thought-out. My only major comments are regarding the interpretation of the results in the context of the recordings that were made.

## Specific Comments:

1. When recording from different cortical layers, it seems as though there would be guaranteed differences in neural populations based on recording depths. Some discussion on relationship between recording depth and the neural subtypes/ subspaces outlined in this paper would be interesting and useful to the reader. If different populations are primarily located at different recording depths, what does this say about the bi-laterality of these signals? Conversely, what is the interpretation if there is no spatial relationship?

**Response to specific comment 1:**

Thank you for this suggestion – we had not yet analyzed this question. We have now analyzed the relationship between arm preference and recording depth for all neurons and present the results in Figure S3. The hypothesis that the dedicated component more closely represents the descending output of the network would make the prediction that neurons in deeper layers (where descending cortical output primarily originates) would be biased toward strong arm preference. This prediction was not supported by the new analysis, although we recognize limitations with the indirect approach employed here (i.e., use of relative depth based on reference to the most superficial electrode position). In addition to the figure, the analysis is discussed in lines 375-385.

2. Relatedly, the dimensionality of the neural recordings in this paper is significantly lower than for unilateral recordings made across the surface of M1 and PMd with Utah electrode arrays (Gallego et al. 2020). Does the nature of the recordings in this paper (4 linear probes) lead to to the lower dimensionality? Some discussion of this as compared to arrays that record across the cortical surface would be useful.

**Response to specific comment 2:**

Our dimensionality estimates are on the lower end of the range reported for M1 and PMd data obtained during similar tasks. We believe this is the combined effect of (1) using PCA for the dimensionality reduction model, (2) the data reconstruction method used for estimating dimensionality, and (3) the recording probes we use, which arrange contacts along an axis perpendicular to the cortical surface. We discuss this issue in the Methods, including references to some of the relevant literature on this topic (lines 1008-1020).

3. What analyses, if any, were performed to ensure a single neuron was not recorded across multiple contacts of the linear probes? 100um spacing is rather close, and it is rather plausible the same units are being recorded from multiple contacts (although it is difficult to say for sure, without knowing the size of the contacts on the V-probes).

**Response to specific comment 3:**

During the initial spike sorting process, we visually checked for repeated neurons by looking for obvious coincident spiking on adjacent channels. We recognize that it is possible to miss repeated neurons with this method. As such, we went back through the dataset and removed one member of any pair of neurons with a firing rate correlation above 0.9 (described in the Methods, lines 900-902). This may occasionally remove a unique neuron but does provide a conservative way to ensure that we have not double-logged any neurons. We repeated all the analyses impacted by this change and have updated the manuscript (statistics and figures) to reflect this change. We note that LDA target classification was not repeated since double-logged neurons should contain completely overlapping information and thus not impact classification.

The main analysis that is likely to be affected by double logging is the dimensionality estimation. Our dimensionality estimation technique was effectively predicting neural activity for one neuron from the PCA projection of the rest of the population. Repeated neurons would leak information about the held-out neuron into that PCA projection and provide a small bias towards higher dimensionality. This may also explain the higher dimensionality at Rest reported in the original analyses (relevant to reviewer #2, minor comment 1).

We have also included more thorough details on our recording probe specifications in the Methods (lines 887-888). This may be helpful for other researchers performing similar experiments and analyses.

## Minor Comments:

1. Does the larger dimensionality during rest have something to do with the sparse firing during rest? Why is this expected?

**Response to minor comment 1:**

We did not have any strong *a priori* expectations on this issue; as such, we simply reported what was observed (which was no longer strongly supported after removing potentially repeated neurons). Our best post-hoc explanation is that the neural activity, while not linked to movement, may still have some correlated structure due to its intrinsic connectivity. That activity during Rest is more sparse and unconstrained as compared to the Instruct and Move phases, so as long as the activity in each neuron can still be reasonably predicted from the rest of the population then the estimated dimensionality will be higher. As a toy example, consider two pairs of neurons that are always correlated even during Rest. Upon movement, all four are driven together by some upstream input. This would result in a transition from 2-dimensional to 1-dimensional by our method.

Since monkey W’s Move phase dimensionality was higher than the Rest phase dimensionality after excluding potentially repeated neurons, we have removed the statement of higher dimensionality at Rest.

2. What are the red circles in 7B?

**Response to minor comment 2:**

This has now been specified in the figure caption.

In addition to revisions made in response to reviewer comments, we have added some details to the Methods describing the datasets used for cross-validation (lines 940-942, 968-972, 976-977). Some of these held-out datasets included trials where the stationary hand was held in a different position while the other reached to the same targets (lines 844-847).

**Have all data underlying the figures and results presented in the manuscript been provided?**
Large-scale datasets should be made available via a public repository as described in the *PLOS Computational Biology* [data availability policy](http://journals.plos.org/ploscompbiol/s/data-availability), and numerical data that underlies graphs or summary statistics should be provided in spreadsheet form as supporting information.

Reviewer #1: **No:** I don`t see any link to a repository for the original data sets in the manuscript.

The dataset is “Private for Peer Review” on the Dryad repository. It will be made public when the manuscript has been accepted, but is currently accessible to reviewers using the access link below: <https://datadryad.org/stash/share/RFdqJc_p44_9cgh0843Ad1ybZEvZllo03e5M3fCBoHc>
