# Supplementary material for: Hybrid dedicated and distributed coding in PMd/M1 provides separation and interaction of bilateral arm signals
Source: PLoS Comput Biol. 2021 Nov 22;17(11):e1009615. doi: 10.1371/journal.pcbi.1009615 (PMC8648118; doi:10.1371/journal.pcbi.1009615)
Supplement: Attachment — Submitted filename: ploscb_response_letter_2.docx [file pcbi.1009615.s011.docx]

Dear Drs. Faisal and Marinazzo,

We thank you and the reviewers again for the evaluation and comments, which continue to add great value to the manuscript. We have provided itemized responses to each comment in the same format as the first revisions. The most significant change is a new paragraph in the discussion section on whole-body control, which discusses bilateral anticipatory adjustments in posture and reflex gain, as well as the likely involvement of cortico-reticulospinal pathways in mediating such effects.

We look forward to your evaluation of the new revisions.

Sincerely,
Tanner Dixon

***********************

**The original reviewer comments are listed below with our responses and revisions described in the red text. Referenced line numbers refer to the “clean” copy of the manuscript submission.**

# **Reviewer #1:**

The authors have responded positively to the previous reviews, and the article is improved. Nevertheless, I have a few further comments about this revised draft.

1. I greatly appreciate the new section that the authors added (lines 665-714) about whole-body control and the possibility that a significant portion of the “distributed” activity might be implicated in control of axial and ipsilateral muscles and body parts to deal with the inevitable biomechanical interactions among multiple body parts during unilateral arm movements. I was a bit disappointed that they spend most of the third paragraph trying to discount this as the main origin for that distributed activity. For instance, one of their arguments is that postural effects should be most relevant during movement, which is reasonable, but they recorded that distributed activity even during the delay period. Anticipatory postural adjustments long before the onset of movements in delay tasks is a well-known phenomenon that has been extensively studied in locomotion and postural perturbation studies. I suppose that for people with a strong computational orientation, a “shared distributed computational subspace” might be sexier than boring old biomechanical compensation, but to others like me, neurophysiological mechanisms of whole-body coordination are also quite sexy! :-)
   a. We thank the reviewer for highlighting the anticipatory nature of postural adjustments, something we recognize but didn’t highlight adequately in the manuscript. We elaborate on this in our response to the following comment. Here we want to emphasize that our intent in the third paragraph was to acknowledge evidence that bilateral muscular control is unlikely to account for the entirety of bilateral cortical signals reported throughout the literature, but not to make strong claims about the main origin. We have revised this section to make that clear (see lines 684-686).
2. Further on this point and on their later discussion section on bilateral signals (lines 754-798), this discussion almost completely ignores the role of the descending cortico-rubral and cortico-reticular projections that are strongly implicated in postural control and bilateral coordination (except lines 746-748). These neural populations might be contributing to the activity in that distributed neural space.
   a. We wholeheartedly agree with this point and have expanded our discussion on whole-body control to include reference to the cortico-reticular pathway and bilateral reticulospinal projections. We discuss the relevance of these pathways for anticipatory postural adjustments and the related topic of reflex modulation. We have modified the text to describe how the presence of activity during the instructed delay may be impacted by these processes as well. These changes can be found in lines 669-681. We have opted not to discuss the cortico-rubral pathways since our read on them is that they primarily target the contralateral effector and therefore add little to the discussion of ipsi- and bilateral activity.
3. I also greatly appreciate the new figure of PC-space trajectories (Figure S7) that they added at my request. It is disappointing though that they make only one fleeting reference to it in the text. It could be cited several times because it illustrates very nicely a number of their major points, such as the much greater variance accounted for in the dedicated neural activity compared to the distributed activity, and the differential evolution of the dedicated and distributed activity throughout the trial in PMd versus M1 between the native and opposite hand.
   a. We have changed the initial reference to this figure in lines 553-556, first providing an overview and then directing the reader towards the central features of the figure. We now flag this figure at other points where we reference Figure 8 as well.
4. Lines 866-868: This new sentence states that the monkeys started the trials in 1 of 3 different arm positions, either with both hands near the body midline or with one hand near the midline and the positioned more laterally. However, they make almost no further comments about this in this manuscript. Did they pool all neural data across these different starting arm configurations for some of the analyses in this manuscript? For instance, did they generate the velocity traces in Figure 1 from just one starting configuration or pooled across all configurations? I can find one statement that they only used the data from trials with both hands close to the start position for dimensionality reduction (lines 1005-1006). They also stated that they did separate analyses for the data from the three starting configurations for Figures 7, 8 and S7 (lines 1000-1001), but it is not clear whether the results shown in those figures were from only one of the configurations, or pooled across all three. This is very confusing. They state in their response to reviewer comments that they plan to report in more detail the effect of arm starting position on neural activity. I think that they have to make some sort of statement about this potential effect and what impact it might have on the results they are presenting in this manuscript. Does it introduce a confound in the interpretation of the present findings?
   a. We have clarified how the different starting configurations were used in each relevant analysis in the methods. The reviewer is correct that, without tailoring the analyses properly with regards to the starting configurations, certain analyses could have bias introduced. We have included more details and discussion on how we have used these datasets while avoiding introduction of such confounds in lines 850-851, 976-988, 1083-1087.
5. Figure 7 and associated text: for some reason, the authors decided to present data in terms of “left arm” and “right arm” instead of ipsilateral and contralateral arm. However, they never explain why they made that change just for that figure, in the Methods (lines 962-964) or anywhere else. Why did they do that?
   a. The models in this figure were trained using data from both hemispheres of the brain. As such, we cannot specify ipsilateral or contralateral, but rather refer to the moving arm as left or right. We have modified the figure legend to make this point.
6. Lines 202, 231-232: Figure 4 no longer has a part 4C.
   a. These references have been replaced with Figure 4B, which corresponds to the arm preference histograms.

# **Reviewer #2:**

I believe the authors have appropriately addressed each of my concerns. I appreciate the extra analysis regarding the electrode depth. While there was no consistent relationship, as the authors pointed out this could be the effect of the recording limitations. I believe it would be of great interest to look at this in the future if/when these limitations are removed.
